# Systemic lipid peroxidation profile from patients with breast cancer changes according to the lymph nodal metastasis status

**DOI:** 10.18632/oncoscience.550

**Published:** 2022-02-24

**Authors:** Stefania Tagliari de Oliveira, Monica Pavaneli Bessani, Thalita Basso Scandolara, Janaína Carla Silva, Aedra Carla Bufalo Kawassaki, Pâmella Aparecida Ferreira Fagotti, Vitor Teixeira Maito, Janoário Athanazio de Souza, Daniel Rech, Carolina Panis

**Affiliations:** ^1^Laboratory of Tumor Biology, State University of West Paraná, Unioeste, Francisco Beltrão, Paraná, Brazil; ^2^Federal University of Rio de Janeiro, Rio de Janeiro, Brazil; ^3^Francisco Beltrão Cancer Hospital, Ceonc, Francisco Beltrão, Paraná, Brazil; ^4^State University of Londrina, Londrina, Paraná, Brazil

**Keywords:** breast cancer, lipid peroxidation, prognosis, lymph nodal metastasis, tumor subtypes

## Abstract

Metastasis is the leading cause of cancer death. Considering that lymph nodes are the major pathway for cancer spreading and that the metastatic process is under oxidative stress effects, this study aims to evaluate the differential lipid peroxidation profile in the blood of breast cancer patients regarding their lymph nodal status (LN). A total of 105 women diagnosed with breast cancer were included before chemotherapy started. LN was determined by assessing the histopathological analysis of patients’ biopsies, and groups were categorized according to the presence (LN+, *n* = 48) or absence (LN−, *n* = 57) of metastases. Lipid peroxidation profiles (LPO) were determined in blood by high-sensitivity chemiluminescence. After patients’ categorization in groups according to their clinicopathological features, LN− patients aged over 50 years presented significantly lower LPO when compared to those under 50 years. Further, LN− patients carrying HER2 positive tumors presented augmented LPO when compared to patients bearing luminal B or triple-negative tumors. LN+ group also had reduced LPO when presented intratumoral clots. The significant contribution of this study was to show that LPO correlates with specific clinical features of patients with breast cancer according to their LN status and that such profile is significantly affected by the presence of metastases.

## INTRODUCTION

Metastasis is responsible for more than 90% of cancer deaths, the reason why scientists have focused on understanding the mechanisms involved in cancer spreading [1].

Breast cancer is the most common malignant neoplasia worldwide, and lymph nodal metastasis is a significant predictor of disease survival, affected by several cellular and molecular events related to chronic inflammation, including oxidative stress [2].

Redox related-events are linked to breast cancer from its rising to progression [3], and the levels of reactive species (RS) have been commonly assessed in such patients by analyzing several products in the blood. Most studies have focused on assessing the pro-oxidative systemic profile of breast cancer patients by measuring the action of free radicals on lipids, a process known as lipid peroxidation. The role of lipid peroxidation in breast cancer has been extensively studied and is intrinsically linked to disease risk [4], treatment aspects [5], and poor prognosis [3].

The most known lipid peroxidation metabolites investigated in breast cancer are malondialdehyde [6, 7], 4-hydroxynonenal [7], and isoprostanes [3], and more recently, hydroperoxides [5, 6]. Hydroperoxides are a group of substances derived from fatty acids, which are enrolled in chemical reactions triggered by free radicals. The lipid hydroperoxides are non-radical metabolites resulting from the propagative lipid peroxidation process that participates in redox processes, frequently associated with cellular damage. These substances are implicated in oxidative stress signaling by directly affecting critical pathways involved in cancer such as cell survival, protein kinases activity, and simulation of natural signal transduction [8], all described as biological processes capable of affecting cancer cell spreading.

In metastasis, fatty acid oxidation is directly implicated in pivotal events that direct disease spreading, as the regulation of cancer stem cells behavior [9]. However, no studies have focused on understanding the role of lipid peroxidation and its metabolites in disease spreading for lymph nodes, especially when measuring hydroperoxides.

Considering the role of oxidative stress in breast cancer progression and that the initial step for disease spreading is triggered in the lymph nodal colonization, the present study aimed to investigate the systemic profile of lipid peroxides in breast cancer patients according to their lymph nodal status concerning the presence of metastases. To reach this goal, we used the high-sensitivity chemiluminescence approach and correlated the results with the main clinicopathological parameters that determine disease prognosis.

## RESULTS

Table 1 shows the clinicopathological characteristics of patients and tumors. A total of 105 women diagnosed with breast cancer were included in this study. For both LN+ and LN− groups, the age of the patients was mainly over 50 years, were overweight, in menopause, with the predominance of tumors of worst prognosis (Luminal B and Triple-negative) had low/intermediate grade tumors. LN− presented predominantly intratumoral clots, while LN+ had tumor predominantly without clots inside.

**Table 1:** Clinicopathological data of patients and tumors

	LN−	LN+
Number of individuals	*N* = 57 (54,28%)	*N* = 48 (45,71%)
Age at diagnosis		
Under 50 years	26,31%	43,75%
Over 50 years	73,68%	56,25%
Body mass index (BMI)		
Eutrophic	24,56%	20,83%
Overweight/obese	61,40%	64,58%
Menopausal status		
No	21,05%	29,16%
Yes	71,92%	60,41%
Molecular subtypes of breast		
Luminal A	14,03%	20,83%
Luminal B	26,31	31,25%
Luminal HER 2	5,2%	4,16%
HER 2	12,28%	8,33%
Triple negative tumors	24,56%	20,83%
Histological grade		
Low/intermediate	78,94%	68,75%
High	17,54%	31,25%
Ki67 index		
Low ki 67	26,31%	33,3%
High ki 67	56,14%	56,25%
Clots		
No	73,68%	29,16%
Yes	22,80%	68,75%

Abbreviations: LN−: negative lymphnodal commitment; LN+: presence of lymphnodal metastasis; HER2: human epidermal growth factor receptor 2.

**Figure 1:**
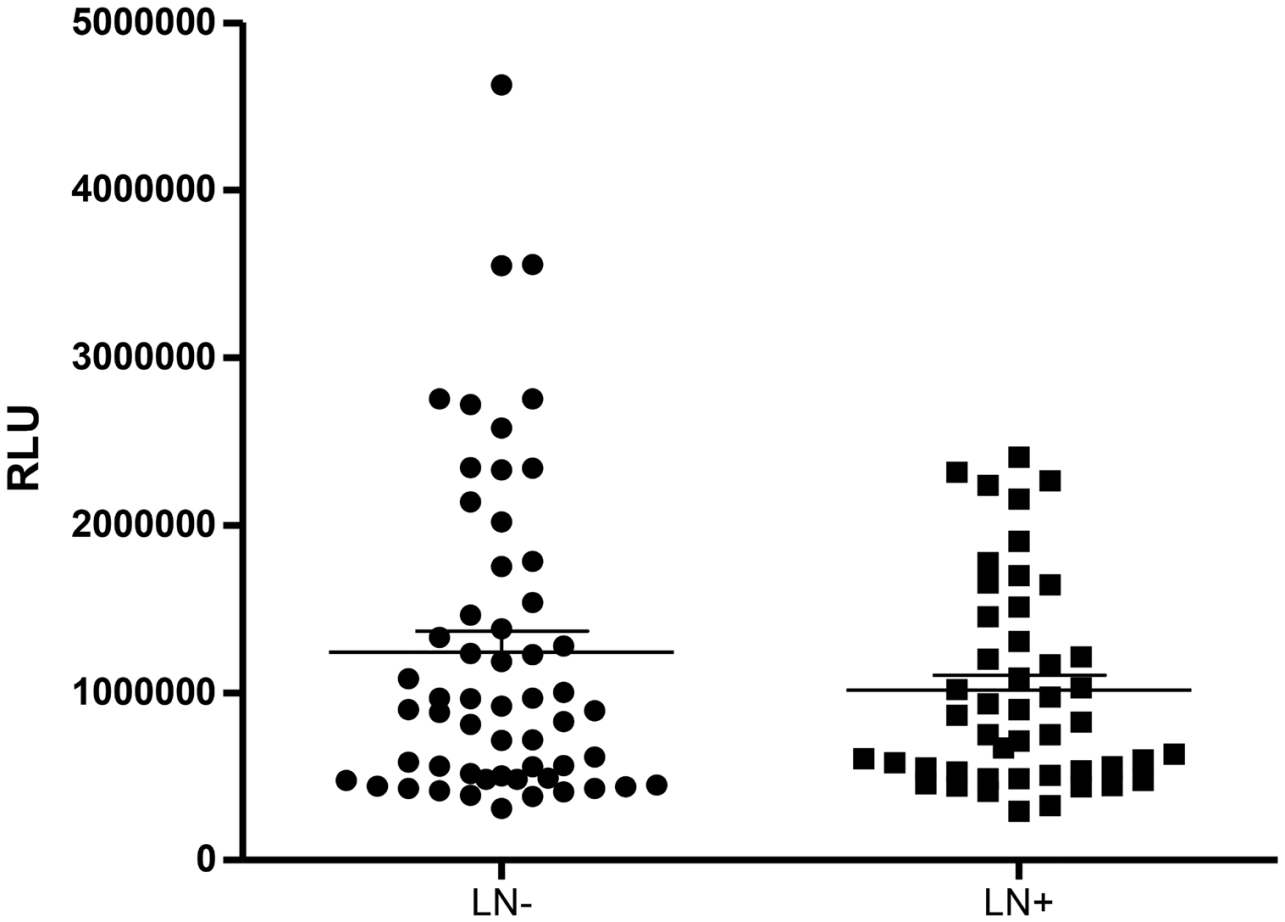
Lipid peroxidation levels in plasma of breast cancer patients according to their lymphonodal status. Abbreviations: LN−: negative lymphnodal commitment; LN+: presence of lymphnodal metastasis; RLU: Relative light unities. ^*^indicates statistical significance, *p* < 0,05.

The distribution of the general plasmatic lipid peroxidation profiling of breast cancer patients regarding their lymphnodal status is shown in Figure 1. No differences were observed in their general profiling of hydroperoxides when comparing both LN negative (LN−) and metastatic LN (LN+) patients (1240906 ± 128293 RLU for LN negative and 1016936 ± 88771 RLU for metastatic LN patients, *p* = 0.5729).

Because of this, we decided to categorize patients from each group according to their clinicopathological features.

**Figure 2:**
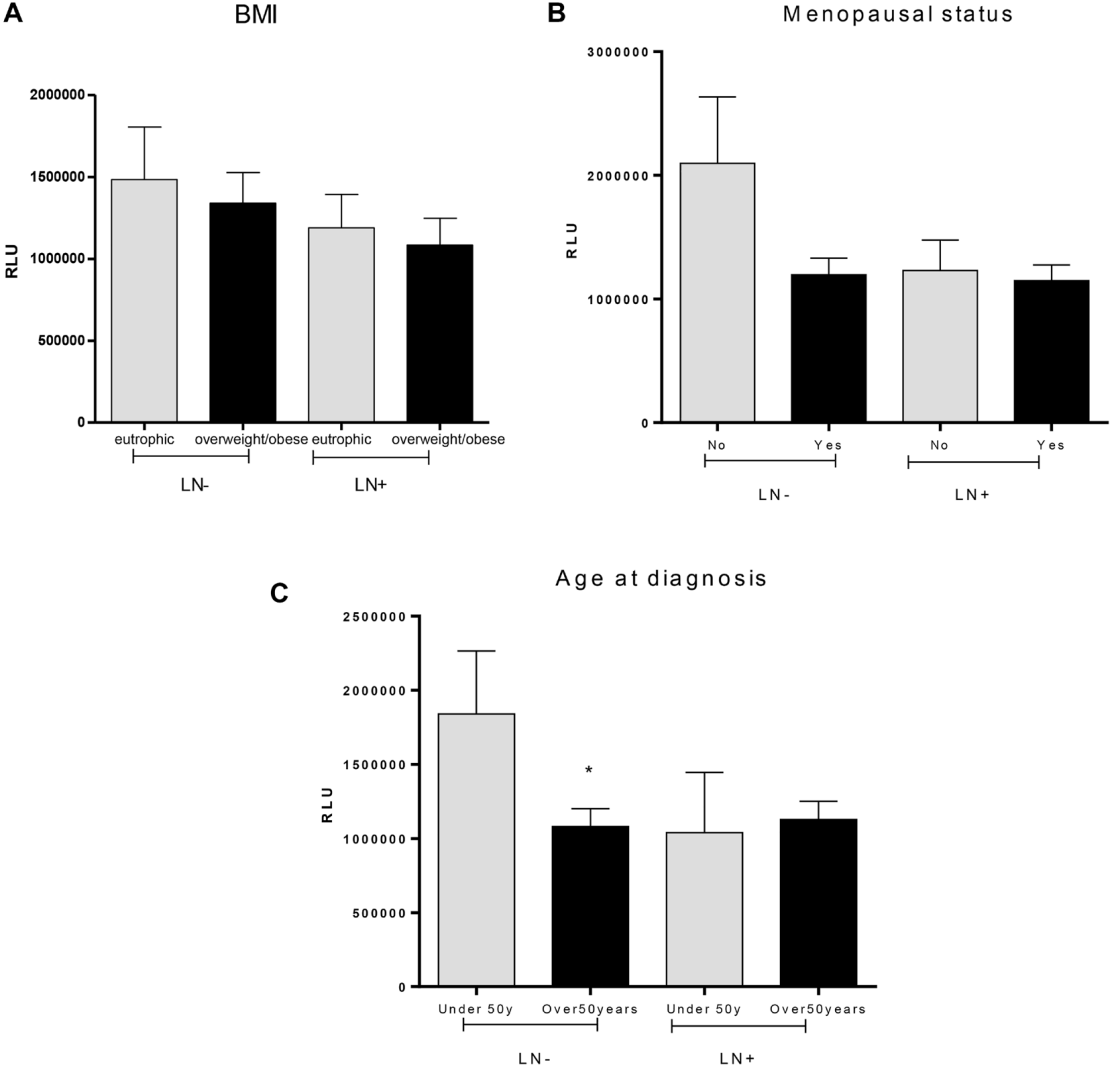
Distribution of lipid peroxidation levels in plasma of breast cancer patients with or without lymphonodal metastasis and its relation with body mass index (**A**), menopausal status (**B**) and age at diagnosis (**C**). Abbreviations: LN−: negative lymphnodal commitment; LN+: presence of lymphnodal metastasis; RLU: Relative light unities; BMI: body mass index. ^*^indicates statistical significance, *p* < 0,05.

Evaluation of lipid peroxidation profiles regarding BMI (Figure 2A) did not show any difference among groups (1483863 ± 319973 RLU for eutrophic LN−, 1338940 ± 188073 RLU for overweight/obese LN−, *p* = 0.6776, 1189774 ± 202844 RLU for eutrophic LN+ and 1084599 ± 162829 RLU for overweight/obese LN+, *p* = 0.7008; *p* = 0.9840 for the comparison between eutrophic LN− versus LN+ and *p* = 0.3208 for the comparison between overweight/obese LN− and overweight/obese LN+).

Further, lipid peroxidation profile was not different for any group of patients regarding their menopausal status, as shown in Figure 2B (2098133 ± 535364 RLU for LN− patients before menopause, 1195384 ± 135427 RLU for LN− patients after menopause, *p* = 0.2694, 1229671 ± 247091 RLU for LN+ patients before menopause and 1147778i ± 128006 RLU for LN+ patients after menopause, *p* = 0.7471).

Significant differences were detected when comparing breast cancer patients concerning their age at diagnosis (Figure 2C) and lipid peroxidation profile inside the LN− group (1840504 ± 423975 RLU for women under 50 years and 1079917 ± 122240 RLU for women over 50 years, *p* = 0.0225). For LN+, no differences were observed (1039094 ± 406345 RLU for women under 50 years and 1128436 ± 122729 RLU for women over 50 years, *p* = 0.8262). When comparing LN− and LN+ groups, no differences were observed regarding this parameter. Comparison of age at diagnosis between LN− and LN+ showed no difference in lipid peroxidation levels (*p* = 0.3514), and there were no differences when comparing patients with late-onset from LN− and LN+ groups (*p* = 0.7802).

**Figure 3:**
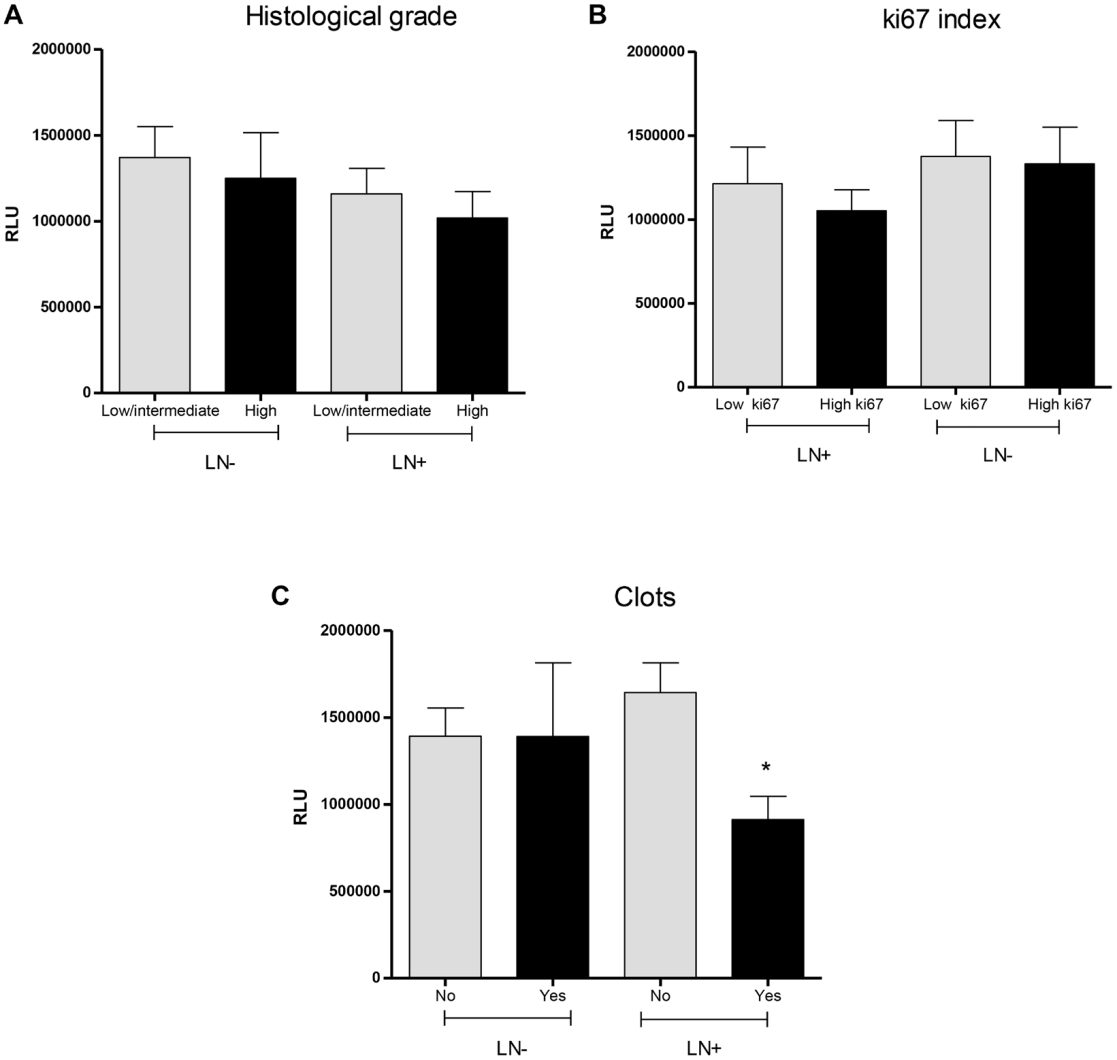
Categorization of lipid peroxidation levels in plasma of breast cancer patients with or without lymphonodal metastasis according to the histological grade (**A**), ki67 index (**B**) and the presence of intratumoral clots (**C**) in tumor biopsies. Abbreviations: LN−: negative lymphnodal commitment; LN+: presence of lymphnodal metastasis; RLU: Relative light unities. ^*^indicates statistical significance, *p* < 0,05.

Other tumor parameters that did not show significant variations regarding lymph nodal status were histological tumor grade (Figure 3A, values to LN negative group: 1370931 ± 178390 RLU for low/intermediate grades and 1248739 ± 267210 RLU for high grade, *p* = 0.8009, and 1159583 ± 148415 RLU for low/intermediate grades and 1019030 ± 153479 RLU for high-grade tumors in LN+ women, *p* = 0.5779) and ki67 index (Figure 3B, 1213006 ± 218513 RLU for LN− and low ki67, 1050851 ± 125526 RLU for LN− and high ki67, *p* = 0.4974; 1374334 ± 215776 RLU for LN+ and low ki67 and 1331162 ± 218590 RLU for LN+ and high ki67, *p* = 0.8922). The comparisons between LN−/low ki67 and LN+/low ki67 and LN−/high ki67 and LN+/high ki67 did not retrieve any significant result (*p* = 0.6056 and 0.8317, respectively).

Patients with metastatic LN presenting intratumoral clots (Figure 3C) showed reduced levels of lipid peroxidation in comparison to the other groups (1391773 ± 161730 RLU for LN− without clots, 1389679 ± 423779 RLU for LN− with clots, 1642296 ± 170810 RLU for LN+ without clots and 911906 ± 133998 RLU for LN+ with clots, *p* = 0.030).

**Figure 4:**
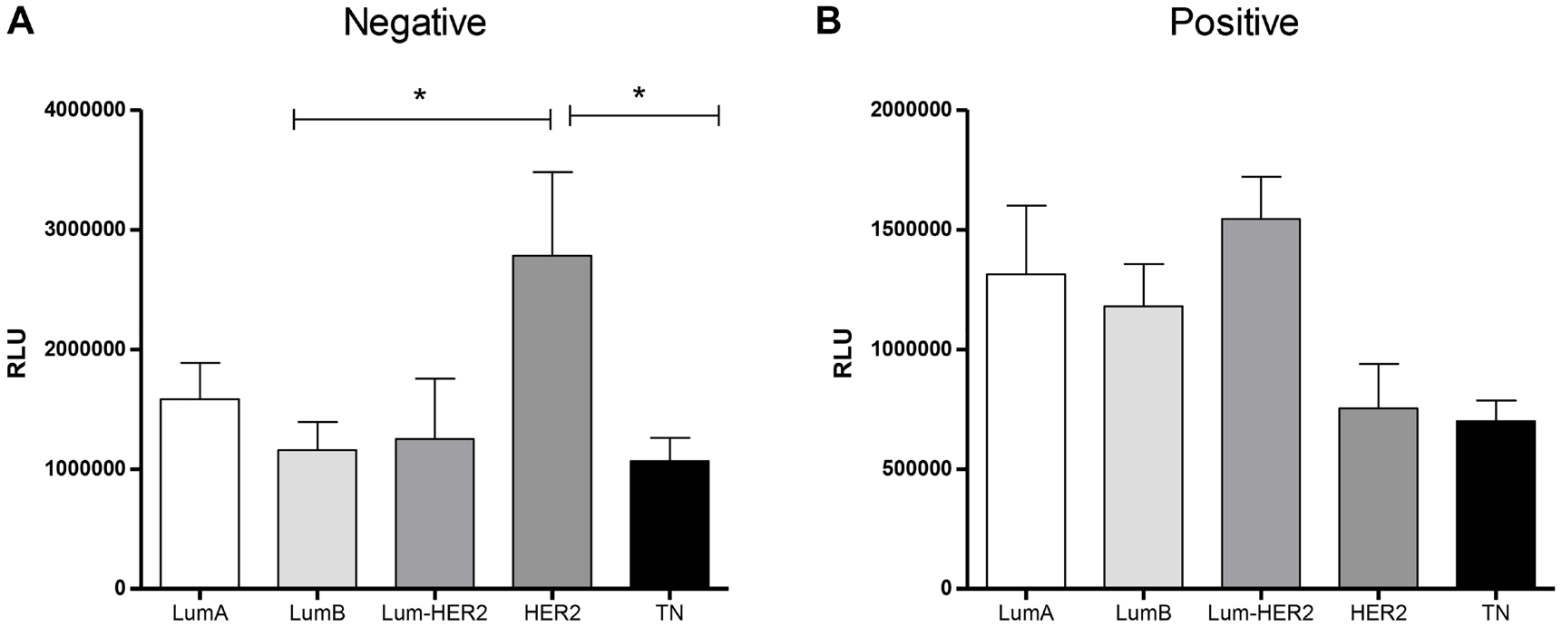
Molecular subtypes of breast cancer and lipid peroxidation levels in plasma of patients negative (**A**) or positive (**B**) to lymphonodal metastasis. Abbreviations: LN−: negative lymphnodal commitment; LN+: presence of lymphnodal metastasis; RLU: Relative light unities; LumA: luminal A tumors; LumB: luminal B tumors; Lum-HER2: luminal tumors with amplification of the receptor of the epidermal growth factor-2; HER2: tumors with amplification of the receptor of the epidermal growth factor 2; TN: triple negative tumors. ^*^indicates statistical significance, *p* < 0,05.

Figure 4 shows the lipid peroxidation levels in plasma concerning the molecular subtypes of breast tumors in both LN− and LN+ groups. As observed in Figure 4A, LN− patients bearing HER2-amplified tumors exhibited the greater levels of lipid peroxides when compared to those with luminal B (*p* = 0.0108) and triple-negative (*p* = 0.060) breast cancer (1583200 ± 303356 RLU for Luminal A, 1159332 ± 233005 RLU for Luminal B, 1249340 ± 507052 RLU for Luminal-HER2, 2782307 ± 697733 RLU for HER2, 1068190 ± 193395 RLU for triple-negative). For LN+ patients (Figure 4B), no differences were observed (1313216 ± 286953 RLU for luminal A, 1179161 ± 176293 RLU for Luminal B, 1544779 ± 175516 RLU for Luminal-HER, 752903 ± 186364 RLU for HER2-amplified and 699464 ± 86230 RLU for triple negatives; *p* > 0.05 for all comparisons).

## DISCUSSION

Metastasis is the leading cause of death in cancer patients, independent of disease topography. For breast cancer, local and distant metastasis are both causes of high lethality [10], but few studies have focused on understanding the putative mechanisms that affect lymph nodal spreading of cancer and its correlation with patients’ prognosis. Multiple mechanisms are involved in cancer spreading, and lymph nodal metastasis is one of the main ways cancer cells reach either the surrounding or distant tissues [11]. For this reason, the present study aimed to investigate if the systemic levels of lipid peroxides in such patients could relate to clinical parameters that predict disease prognosis.

Our data showed for the first time that the levels of lipid peroxidation in plasma vary in women with breast cancer according to their specific clinicopathological features regarding lymph nodal metastasis. Main findings in patients without lymph nodal metastasis included significantly higher lipid peroxidation levels in those under 50 years and carrying HER2 amplified tumors. In patients presenting lymph nodal metastasis, we found reduced lipid peroxidation levels in those exhibiting intratumoral clots.

It is well established that inflammatory mediators are essential in cancer spreading, including oxidative stress-derived products [12]. Lipid peroxidation is a biological process resultant from a wide of cellular reactions that produce free radicals capable of attacking its lipidic content [13], and lymph nodes can be potentially affected in this context.

Studies have described that the lipid peroxidation status of breast cancer patients correlates with the primary tumor mass [14] and is enhanced in patients with advanced disease [15]. The group of patients without lymph nodal metastasis had more significant heterogeneity in their levels of peroxides when compared to those with metastasis. In this way, a recent study demonstrates that the lipid peroxidation profile of sentinel lymph nodes removed from patients with breast cancer varies depending on the absence or presence of micro or macrometastases [16]. Therefore, if considering that breast cancer cells are under constant adaptation – aiming to multiplicate and spread [17], the dispersion of data observed could be a consequence of possible transformations that were occurring systemically in each LN− patient, such as epithelial to mesenchymal transition of tumor cells and immune edition, well known as generators of oxidative stress [18, 19].

We also evaluated classical risk factors that affect breast cancer risk and progression regarding lipid peroxidation profiling and lymph nodal status in the studied patients. BMI, menopause, and age at diagnosis are known factors that directly affect disease risk [20]; therefore, we investigated if lipid peroxidation status was differentially expressed according to such parameters. Despite lipid peroxidation metabolites being positively associated with the risk of developing breast cancer in overweight women [21], our data showed that patients carrying early-stage breast cancer had no difference in this parameter, regardless of their lymph nodal status. The same was observed concerning the menopausal status of these patients. It is discussed that menopause can imbalance the redox status of women with breast cancer [22], despite evidence reporting no differences between pre and post-menopausal patients [23], as reported here.

LN− patients showed a meaningful difference in lipid peroxidation status when considering their early or late age at diagnosis. Patients over 50 years at diagnosis displayed reduced levels of hydroperoxides when compared to younger patients. Although no studies reporting the lipid peroxidation status in breast cancer women considering their age at diagnosis were found, our findings follow recent evidence that reported a positive relationship between telomere length and urinary levels of lipid peroxidation metabolites in healthy women [24]. Further, results presented here indicate that the presence of lymph nodal metastasis seems to nullify the differences observed concerning the age of patients.

A significant reduction of hydroperoxides was observed in LN+ patients presenting intratumoral clots, and two factors presented here can help to understand this result. Firstly, circulating tumor cells favor cancer metastasis in breast cancer [25] by affecting the local hemostasis. Secondly, thrombotic disorders are conjoint with cancer and inflammation, and oxidative stress is a phenomenon encountered under these conditions that affect the coagulation cascade [26]. Since lipid peroxides generated by activated platelets can trigger thrombus formation [27], the association of such mechanisms could be a plausible multi-step mechanism present in LN+ patients with breast cancer.

In the last decades, the scientific community has been interested in understanding the biological factors that help explain the clinical behavior of the molecular subtypes of breast cancer, including oxidative stress-related mediators [28]. To the best of our knowledge, this is the first report investigating the systemic lipid peroxidation status and its correlation with the different breast cancer molecular subtypes.

Only patients without lymph nodal metastasis exhibited differential expression of hydroperoxides in blood when comparing their tumor subtype. HER2 patients had the highest levels of lipid peroxidation in comparison to both luminal B and triple-negative ones. Although only two studies have switched on lipid peroxidation metabolites and breast cancer molecular subtypes, such substances have been positively associated with estrogen receptors expression [29] when evaluated in urine and found in high levels in the blood of patients bearing aggressive subtypes of breast cancer [30]. In the study of Ferroni and colleagues, breast cancer patients carrying HER2 tumors are reported with increased levels of urinary oxidative stress markers compared to both luminal and triple-negative patients, similarly, as the data reported here to LN− patients.

A significant shortcoming of our study includes the fact that we did not have access to the sentinel lymph nodal status of patients and the lack of *in situ* analyses of lipid peroxidation of lymph nodes affected or not by metastasis. In addition, scarce literature on this topic difficult its clinical interpretation.

The significant contribution of this study was to show that lipid peroxidation correlates with specific clinical features of patients with breast cancer according to their lymph nodal status and that such profile is significantly affected by the presence of metastases.

## MATERIALS AND METHODS

### Patients and samples

A total of 105 women diagnosed with breast cancer attended at Francisco Beltrão Cancer Hospital (CEONC), Paraná, Brazil, over the period from May 2015 to December 2017 were investigated. Only patients with complete medical records and kept in clinical follow-up in our Institution were included in the study.

This research was approved by the Institutional Ethics Committee and is registered by the authorization number 35524814.4.0000.0107. All participants signed consent terms, and the study was conducted following the Helsinki Declaration. Inclusion criteria included female patients diagnosed with unilateral ductal carcinoma of the breast, bearing stage II/operable disease that had available data about the lymph nodal status.

**Figure 5:**
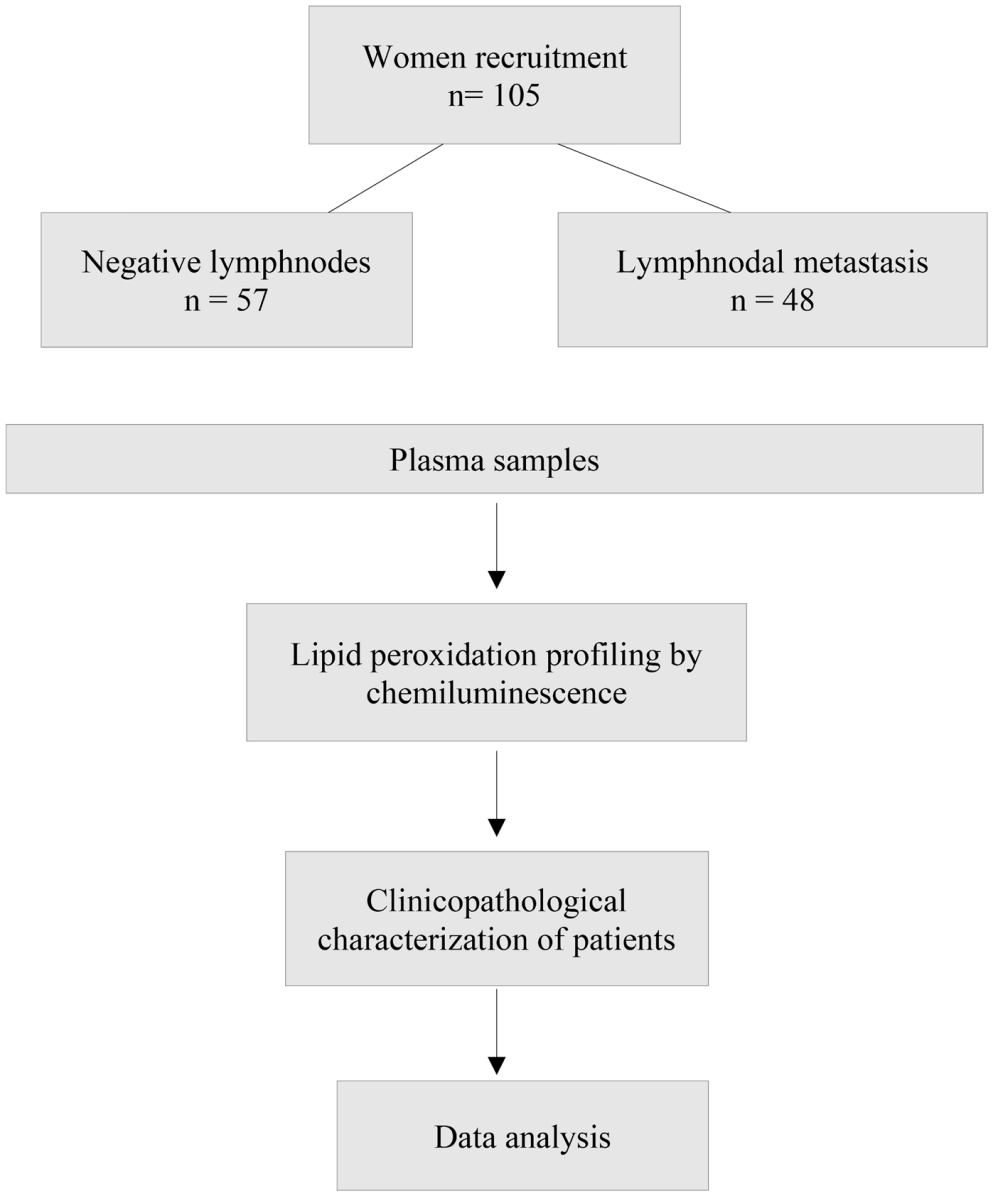
Design of the study.

This study is a prospective study, and the design of the study is shown in Figure 5. Peripheral blood samples were collected by venous punction in EDTA tubes (5 mL), centrifuged at 4000 rpm, and plasma aliquots frozen until analyses.

Lymph nodal status was assessed based on the analyses of biopsies obtained at the surgery by a pathologist. Clinical records were assessed for the obtention of clinical data, including age at diagnosis, body mass index, menopausal status, histological grade of tumors, and the presence of intratumoral clots. Molecular subtypes of breast tumors were determined by immunohistochemistry. The results were categorized as Luminal A (positive to estrogen and/or progesterone receptors, with a ki67 index lower than 14%), Luminal B (positive to estrogen and/or progesterone receptors, with a ki67 index higher than 14%), Luminal-HER2 (positive to estrogen and/or progesterone receptors, with amplification of the receptor of the epidermal growth factor 2 – HER2, and any ki67 index), HER2 amplified (with amplification of the receptor of the epidermal growth factor 2 – HER2, and any ki67 index) and triple-negative (negative for estrogen, progesterone, and HER2 receptors, with any ki67 index).

### Determination of lipid peroxidation profiles

Plasmatic levels of or hydroperoxides were estimated by measuring hydroperoxides levels, employing the high sensitivity chemiluminescence method as previously described [5]. Briefly, an aliquot of plasma (125 μL) was added to a microtube containing 865 μL of disodium monobasic phosphate buffer 0.1M pH 8.5 and 20 μL of tert-butyl hydroperoxide (3 mM). The reaction was monitored for 40 minutes, one read/minute in the kinetic mode, in a Glomax luminometer (Promega, USA). The results were expressed as relative light unities (RLU), and the entire curve was analyzed to integrate the area under the curve in the software OriginLab 9.0.

### Data analysis

The Grubbs test was performed to detect putative outliers. No outliers were detected in this study. Data were expressed as means ± errors of the means, and the results were compared by Mann-Whitney (non-parametric data) or Student’s *t*-test (parametric data). ANOVA test was also performed when comparing more than two groups. All statistical analyses were performed using GraphPad Prism version 7.0 (GraphPad Software, San Diego, USA). A value of *p* < 0.05 was considered significant.

## Abbreviations

LN: Lymphnode
RLU: Relative light unities
LumA: luminal A tumors
LumB: luminal B tumors
Lum-HER2: luminal tumors with amplification of the receptor of the epidermal growth factor-2
HER2: tumors with amplification of the receptor of the epidermal growth factor 2
TN: triple negative tumors
BMI: body mass index

## References

[1] Ding J, Jiang L, Wu W. Predictive Value of Clinicopathological Characteristics for Sentinel Lymph Node Metastasis in Early Breast Cancer. Med Sci Monit. 2017; 23:4102–8. https://doi.org/10.12659/msm.902795. 28839123 10.12659/MSM.902795 PMC5584843

[2] Carioca AA, Verde SM, Luzia LA, Rondó PH, Latorre MR, Ellery TH, Damasceno NR. Association of oxidative stress biomarkers with adiposity and clinical staging in women with breast cancer. Eur J Clin Nutr. 2015; 69:1256–61. https://doi.org/10.1038/ejcn.2015.84. 26039316 10.1038/ejcn.2015.84

[3] Lee JD, Cai Q, Shu XO, Nechuta SJ. The Role of Biomarkers of Oxidative Stress in Breast Cancer Risk and Prognosis: A Systematic Review of the Epidemiologic Literature. J Womens Health (Larchmt). 2017; 26:467–82. https://doi.org/10.1089/jwh.2016.5973. 28151039 10.1089/jwh.2016.5973 PMC5446608

[4] Boyd NF, McGuire V. The possible role of lipid peroxidation in breast cancer risk. Free Radic Biol Med. 1991; 10:185–90. https://doi.org/10.1016/0891-5849(91)90074-d. 1864523 10.1016/0891-5849(91)90074-d

[5] Panis C, Herrera AC, Victorino VJ, Campos FC, Freitas LF, De Rossi T, Colado Simão AN, Cecchini AL, Cecchini R. Oxidative stress and hematological profiles of advanced breast cancer patients subjected to paclitaxel or doxorubicin chemotherapy. Breast Cancer Res Treat. 2012; 133:89–97. https://doi.org/10.1007/s10549-011-1693-x. 21811816 10.1007/s10549-011-1693-x

[6] Wang C, Yu J, Wang H, Zhang J, Wu N. Lipid peroxidation and altered anti-oxidant status in breast adenocarcinoma patients. Drug Res (Stuttg). 2014; 64:690–92. https://doi.org/10.1055/s-0034-1372580. 25050522 10.1055/s-0034-1372580

[7] Szychta P, Zadrozny M, Lewinski A, Karbownik-Lewinska M. Increased oxidative damage to membrane lipids following surgery for breast cancer. Neuro Endocrinol Lett. 2014; 35:602–7. 25617883

[8] Girotti AW. Lipid hydroperoxide generation, turnover, and effector action in biological systems. J Lipid Res. 1998; 39:1529–42. 9717713

[9] Yi M, Li J, Chen S, Cai J, Ban Y, Peng Q, Zhou Y, Zeng Z, Peng S, Li X, Xiong W, Li G, Xiang B. Emerging role of lipid metabolism alterations in Cancer stem cells. J Exp Clin Cancer Res. 2018; 37:118. https://doi.org/10.1186/s13046-018-0784-5. 29907133 10.1186/s13046-018-0784-5 PMC6003041

[10] Geng SQ, Alexandrou AT, Li JJ. Breast cancer stem cells: Multiple capacities in tumor metastasis. Cancer Lett. 2014; 349:1–7. https://doi.org/10.1016/j.canlet.2014.03.036. 24727284 10.1016/j.canlet.2014.03.036 PMC4177877

[11] Seyfried TN, Huysentruyt LC. On the origin of cancer metastasis. Crit Rev Oncog. 2013; 18:43–73. https://doi.org/10.1615/critrevoncog.v18.i1-2.40. 23237552 10.1615/critrevoncog.v18.i1-2.40 PMC3597235

[12] Ayala A, Muñoz MF, Argüelles S. Lipid peroxidation: production, metabolism, and signaling mechanisms of malondialdehyde and 4-hydroxy-2-nonenal. Oxid Med Cell Longev. 2014; 2014:360438. https://doi.org/10.1155/2014/360438. 24999379 10.1155/2014/360438 PMC4066722

[13] Niki E. Lipid peroxidation: physiological levels and dual biological effects. Free Radic Biol Med. 2009; 47:469–84. https://doi.org/10.1016/j.freeradbiomed.2009.05.032. 19500666 10.1016/j.freeradbiomed.2009.05.032

[14] Herrera AC, Victorino VJ, Campos FC, Verenitach BD, Lemos LT, Aranome AM, Oliveira SR, Cecchini AL, Simão AN, Abdelhay E, Panis C, Cecchini R. Impact of tumor removal on the systemic oxidative profile of patients with breast cancer discloses lipid peroxidation at diagnosis as a putative marker of disease recurrence. Clin Breast Cancer. 2014; 14:451–59. https://doi.org/10.1016/j.clbc.2014.05.002. 25077997 10.1016/j.clbc.2014.05.002

[15] Panis C, Victorino VJ, Herrera AC, Freitas LF, De Rossi T, Campos FC, Simão AN, Barbosa DS, Pinge-Filho P, Cecchini R, Cecchini AL. Differential oxidative status and immune characterization of the early and advanced stages of human breast cancer. Breast Cancer Res Treat. 2012; 133:881–88. https://doi.org/10.1007/s10549-011-1851-1. 22048816 10.1007/s10549-011-1851-1

[16] Ramírez-Expósito MJ, Urbano-Polo N, Dueñas B, Navarro-Cecilia J, Ramírez-Tortosa C, Martín-Salvago MD, Martínez-Martos JM. Redox status in the sentinel lymph node of women with breast cancer. Ups J Med Sci. 2017; 122:207–16. https://doi.org/10.1080/03009734.2017.1403522. 29264992 10.1080/03009734.2017.1403522 PMC5810224

[17] Yousefi M, Nosrati R, Salmaninejad A, Dehghani S, Shahryari A, Saberi A. Organ-specific metastasis of breast cancer: molecular and cellular mechanisms underlying lung metastasis. Cell Oncol (Dordr). 2018; 41:123–40. https://doi.org/10.1007/s13402-018-0376-6. 29568985 10.1007/s13402-018-0376-6 PMC12995240

[18] Sridaran D, Ramamoorthi G, MahaboobKhan R, Kumpati P. Oxystressed tumor microenvironment potentiates epithelial to mesenchymal transition and alters cellular bioenergetics towards cancer progression. Tumour Biol. 2016; 37:13307–22. https://doi.org/10.1007/s13277-016-5224-6. 27460079 10.1007/s13277-016-5224-6

[19] Suman S, Sharma PK, Rai G, Mishra S, Arora D, Gupta P, Shukla Y. Current perspectives of molecular pathways involved in chronic inflammation-mediated breast cancer. Biochem Biophys Res Commun. 2016; 472:401–9. https://doi.org/10.1016/j.bbrc.2015.10.133. 26522220 10.1016/j.bbrc.2015.10.133

[20] Howell A, Anderson AS, Clarke RB, Duffy SW, Evans DG, Garcia-Closas M, Gescher AJ, Key TJ, Saxton JM, Harvie MN. Risk determination and prevention of breast cancer. Breast Cancer Res. 2014; 16:446. https://doi.org/10.1186/s13058-014-0446-2. 25467785 10.1186/s13058-014-0446-2 PMC4303126

[21] Dai Q, Gao YT, Shu XO, Yang G, Milne G, Cai Q, Wen W, Rothman N, Cai H, Li H, Xiang Y, Chow WH, Zheng W. Oxidative stress, obesity, and breast cancer risk: results from the Shanghai Women’s Health Study. J Clin Oncol. 2009; 27:2482–88. https://doi.org/10.1200/JCO.2008.19.7970. 19380446 10.1200/JCO.2008.19.7970 PMC2684853

[22] Ramírez-Expósito MJ, Sánchez-López E, Cueto-Ureña C, Dueñas B, Carrera-González P, Navarro-Cecilia J, Mayas MD, Arias de Saavedra JM, Sánchez-Agesta R, Martínez-Martos JM. Circulating oxidative stress parameters in pre- and post-menopausal healthy women and in women suffering from breast cancer treated or not with neoadjuvant chemotherapy. Exp Gerontol. 2014; 58:34–42. https://doi.org/10.1016/j.exger.2014.07.006. 25019472 10.1016/j.exger.2014.07.006

[23] Kumaraguruparan R, Kabalimoorthy J, Nagini S. Correlation of tissue lipid peroxidation and antioxidants with clinical stage and menopausal status in patients with adenocarcinoma of the breast. Clin Biochem. 2005; 38:154–58. https://doi.org/10.1016/j.clinbiochem.2004.10.012. 15642278 10.1016/j.clinbiochem.2004.10.012

[24] Erdmann NJ, Harrington LA, Martin LJ. Mammographic density, blood telomere length and lipid peroxidation. Sci Rep. 2017; 7:5803. https://doi.org/10.1038/s41598-017-06036-y. 28725051 10.1038/s41598-017-06036-y PMC5517610

[25] Beinse G, Berger F, Cottu P, Dujaric ME, Kriegel I, Guilhaume MN, Diéras V, Cabel L, Pierga JY, Bidard FC. Circulating tumor cell count and thrombosis in metastatic breast cancer. J Thromb Haemost. 2017; 15:1981–88. https://doi.org/10.1111/jth.13792. 28779538 10.1111/jth.13792

[26] Martinez M, Weisel JW, Ischiropoulos H. Functional impact of oxidative posttranslational modifications on fibrinogen and fibrin clots. Free Radic Biol Med. 2013; 65:411–18. https://doi.org/10.1016/j.freeradbiomed.2013.06.039. 23851017 10.1016/j.freeradbiomed.2013.06.039 PMC3852169

[27] Gorog P, Kovacs IB. Lipid peroxidation by activated platelets: a possible link between thrombosis and atherogenesis. Atherosclerosis. 1995; 115:121–28. https://doi.org/10.1016/0021-9150(94)05506-e. 7669082 10.1016/0021-9150(94)05506-e

[28] Victorino VJ, Barroso WA, Assunção AK, Cury V, Jeremias IC, Petroni R, Chausse B, Ariga SK, Herrera AC, Panis C, Lima TM, Souza HP. PGC-1β regulates HER2-overexpressing breast cancer cells proliferation by metabolic and redox pathways. Tumour Biol. 2016; 37:6035–44. https://doi.org/10.1007/s13277-015-4449-0. 26602383 10.1007/s13277-015-4449-0

[29] Ferroni P, Santilli F, Cavaliere F, Simeone P, Costarelli L, Liani R, Tripaldi R, Riondino S, Roselli M, Davi G, Guadagni F. Oxidant stress as a major determinant of platelet activation in invasive breast cancer. Int J Cancer. 2017; 140:696–704. https://doi.org/10.1002/ijc.30488. 27784132 10.1002/ijc.30488

[30] Herrera AC, Panis C, Victorino VJ, Campos FC, Colado-Simão AN, Cecchini AL, Cecchini R. Molecular subtype is determinant on inflammatory status and immunological profile from invasive breast cancer patients. Cancer Immunol Immunother. 2012; 61:2193–201. https://doi.org/10.1007/s00262-012-1283-8. 22618884 10.1007/s00262-012-1283-8 PMC11028631

